# Platelet and Red Cell Indices: A Potential Biomarker for Predicting Pulmonary Hypertension in Chronic Obstructive Pulmonary Disease (COPD) Patients

**DOI:** 10.7759/cureus.88401

**Published:** 2025-07-21

**Authors:** Kishore Kanna S, Kanabur Thirthashree, V Bhuvaneswari, S Parinita, Archana Baburao, Ashika Adinarayan

**Affiliations:** 1 Department of Pulmonary Medicine, Rajarajeswari Medical College and Hospital, Bengaluru, IND; 2 Department of Pulmonary Medicine, Kempegowda Institute of Medical Sciences, Bengaluru, IND; 3 Department of Respiratory Medicine, Rajarajeswari Medical College and Hospital, Bengaluru, IND

**Keywords:** copd, platelet indices, platelet-to-lymphocyte ratio, pulmonary hypertension, red cell distribution width (rdw), red cell indices

## Abstract

Introduction: Chronic obstructive pulmonary disease (COPD) is a chronic progressive condition marked by inflammation and airflow limitation. Platelets and red blood cells play an important role in the pathogenesis of COPD and pulmonary hypertension (PH) by mediating thrombotic, inflammatory, and immune processes in the lungs. Blood indices, such as platelet-to-lymphocyte ratio (PLR), platelet distribution width (PDW), plateletcrit (PCT), red cell distribution width (RDW), mean platelet volume (MPV), and platelet count (PLT), have emerged as potential biomarkers for predicting the severity of PH. This study compares these indices and clinical profiles between COPD and non-COPD patients.

Objectives: This study aimed to compare the values of platelet indices and red cell indices between COPD and non-COPD patients and to investigate the predictive value of platelet indices and red cell indices for PH in COPD patients.

Methods: This cross-sectional study included 100 participants, categorized into two groups: COPD (n = 75) and non-COPD (n = 25). Laboratory parameters, including platelet and red cell indices, and pulmonary function parameters such as post-bronchodilator FEV1, forced vital capacity, and diffusion capacity of carbon monoxide (DLCO), were analyzed. PH severity (mild, moderate, and severe) was assessed by 2D-echo, and statistical significance was determined using t-tests, chi-square tests, and one-way analysis of variance.

Results: COPD patients had lower lymphocyte counts (1,352.6 ± 483.9 vs. 2,253.4 ± 867.6; p < 0.001), higher PLR (253.8 ± 86.6 vs. 126.5 ± 79.4; p = 0.010), elevated RDW (15.4 ± 0.9 vs. 11.8 ± 1.4; p < 0.001), lower PDW (10.2 ± 1.6 vs. 11.2 ± 1.6), and higher PLT (318,720 ± 99,905.2 vs. 264,500 ± 11,2661.7) compared to non-COPD patients. PLR (p = 0.040), RDW (p <0.001), and DLCO (p = 0.025) predicted PH and increased with the severity of PH, while PDW, MPV, PCT, and PLT did not predict PH.

Conclusion: Blood markers, particularly RDW and PLR, are effective in predicting the severity of PH in COPD patients and can be used in early suspicion and diagnosis of PH in COPD patients, especially in low-resource settings.

## Introduction

Chronic obstructive pulmonary disease (COPD) is a heterogeneous lung condition characterized by chronic respiratory symptoms due to abnormalities of the airways and alveoli [1]. It is a chronic progressive inflammatory condition characterized by marked airflow limitation. Globally, about three million deaths occur due to COPD every year [2].

Pulmonary hypertension (PH), characterized by chronic elevation of pulmonary arterial pressure and pulmonary vascular resistance leading to right heart failure, is a major complication of COPD and is an established independent prognostic factor. PH due to COPD is classified as group 3, which comprises PH caused by lung diseases [3,4]. The prevalence of PH in stable COPD is widely reported to range from 20% to 91% depending on many prospective views, such as PH definition, COPD severity grades, and the methods by which the pulmonary pressure is measured [5,6]. Airflow limitation in COPD patients causes alterations in pulmonary vasculature by intimal hyperplasia and smooth muscle hypertrophy/hyperplasia. Airway remodeling causes increased shear stress in the pulmonary arteries. When combined with vascular injury, this can induce neointimal lesions. Additionally, products from tobacco smoke may directly cause dysfunction in endothelial cells, resulting in impaired release of endothelial nitric oxide synthase and increased expression of vascular endothelial growth factor. These processes contribute to the development of PH in patients with COPD [7].

Platelet activation, either due to systemic inflammation, oxidative stress condition, or hypoxemia, causes a cascade of platelet aggregation leading to the release of its chemical mediators, resulting in endothelial injury [8,9]. The reactive platelet cells contain alpha and delta dense granules. Alpha granules store platelet growth factors, vasoactive substances such as thromboxane A2, and cytokines, such as interleukin (IL)-1 and IL-6, while delta granules store serotonin [10-13]. The injured endothelium acts as a thrombogenic focus, which consequently attracts more platelets and starts to release vasoactive substances, causing pulmonary vasoconstriction, proliferation of smooth muscle cells, and pulmonary remodeling [6].

Ultimately, platelets were involved in the pathophysiology of PH by different mechanisms of action: 1) thrombosis formation due to pulmonary vascular endothelial dysfunction, 2) inflammatory cytokines release, especially IL-6, and 3) serotonin production, which was responsible for neutrophil aggregation, producing various cycles of the inflammatory process [6]. Hence, platelet indices like platelet distribution width (PDW), mean platelet volume (MPV), plateletcrit (PCT), platelet count (PLT), and platelet-to-lymphocyte ratio (PLR) can be useful for predicting PH in COPD patients.

The red cell index, namely red cell distribution width (RDW), has been studied as a biomarker in various cardiovascular conditions, including myocardial infarction and congestive cardiac failure with various etiologies [14-17]. It is associated with defective erythropoiesis, increased red blood cell (RBC) death, or a shorter RBC lifespan [18]. Hence, this study aims to utilize this blood index, along with RBC count and hemoglobin (Hb), as biomarkers for predicting PH in COPD patients.

Aims and objectives

This study aimed to compare platelet and red cell indices between COPD and non-COPD patients and evaluate the predictive value of these hematological markers for the presence and severity of PH in COPD patients.

## Materials and methods

This is a cross-sectional study conducted in the Department of Respiratory Medicine, Rajarajeswari Medical College and Hospital, Bengaluru, affiliated to Dr. M.G.R. Educational and Research Institute, from March 2025 to June 2025. All the willing participants were enrolled in the study after screening for the inclusion and exclusion criteria. Written informed consent was obtained from all subjects, and approval was obtained from the Institutional Ethics Committee (RRMCH-IEC/14/2025).

**Figure 1:**
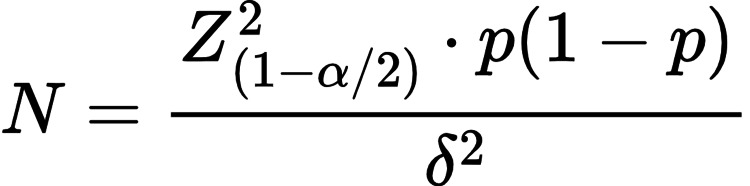
Sample calculation formula

Both male and female participants aged above 40 years, who were able to perform spirometry and who did not have other causes of PH, were included in this study. The COPD group consisted of subjects with a confirmed diagnosis of COPD based on post-bronchodilator FEV1/forced vital capacity (FVC) < 0.70. The non-COPD group consisted of subjects undergoing evaluation for preemployment fitness, fitness for surgery with nonrespiratory systemic complaints, with no history of respiratory symptoms, and normal 2D echocardiographic findings, regardless of their smoking status. Participants with history of hematological disorder or malignancies; with abnormal hematocrit and/or abnormal WBC count; on antiplatelet or anticoagulant drugs in the past 15 days; and who have increased chances of thrombotic events like malignancies, thyroid disorder, embolism, and HIV infection; pregnant women; people unwilling to cooperate; and patients with any relative contraindication for performing spirometry were excluded in our study. The sample size was calculated as 100 participants using the formula in Figure 1.

Here, Z (1 - a/2) =1.96 (for 95% confidence interval), p = 0.074 (based on the previous literature by Daniel et al. [19] who gave a pooled prevalence of COPD as 7.4%), and d (margin of error) = 0.052, which gave a value of N = 97.4. Finally, the value was rounded to 100 participants.

All study participants were subjected to spirometry and diffusion capacity of carbon monoxide (DLCO) according to the Global Initiative for Chronic Obstructive Lung Disease 2025 guidelines [20]. Post-bronchodilator values of forced-expiratory volume in one second (FEV1), FVC, maximal mid-expiratory flow rate (FEF_25-75%_), and DLCO were taken. Transthoracic echocardiography was performed using a 2.5 MHz transducer with patients in the left lateral decubitus position. All echocardiographic assessments were conducted in the cardiology department by experienced sonographers, and the images were reviewed and interpreted by a board-certified cardiologist, in accordance with the European Society of Cardiology (ESC)/European Respiratory Society (ERS) 2022 guidelines [3] for the assessment of PH. Using Bernoulli’s equation, the pressure gradient across the tricuspid valve (TRV) was measured and added to the estimated right atrial pressure (eRAP). The sum of TRV and eRAP gave the pulmonary artery systolic pressure (PASP). Patients were classified as mild (30-50 mmHg), moderate (50-70 mmHg), or severe (>70 mmHg) if their PASP was greater than 30 mmHg. Venipuncture was done from the median cubital vein, and 2 mL of blood was drawn under sterile aseptic precautions; 2 mL of blood was drawn and transported in a K_2_ ethylenediaminetetraacetic acid (15%) vacutainer tube and analyzed in an automated analyzer called Sysmex XN 350 (Sysmex Corporation, Kobe, Japan). Values of platelet indices, namely PDW, MPV, PCT, PLT, and PLR, and red cell indices, including RDW, RBC, and HB, were taken.

Statistical analysis

Statistical analysis was conducted using Excel 2019 (Microsoft Corporation, Redmond, WA) and Jamovi version 2.4.11, jamovi (Version 2.4.11) (Computer Software); retrieved from https://www.jamovi.org. Continuous variables were expressed as mean ± standard deviation, and categorical variables as frequencies and percentages. Independent samples t-tests were used to compare continuous variables between two groups, while one-way analysis of variance was applied for comparisons involving more than two groups. Associations between categorical variables were evaluated using the chi-square test. A p value of less than 0.05 was considered statistically significant.

## Results

A total of 100 patients were included in the study. The mean age was 62.2 ± 12.1 years, with a male predominance (68%). COPD was diagnosed in 75 patients (75%), while 25 patients (25%) were classified as non-COPD. PH was present in 48% of the cohort. Among patients with PH (n = 49), mild PH was the most common (75.5%), followed by moderate (16.3%) and severe PH (18.2%). The majority of the study population (57%) were smokers (as shown in Table 1).

**Table 1:** Descriptive summary of demographics, diagnosis, and severity of PH Variables are presented as mean ± SD for continuous data or as n (%) for categorical data. The table summarizes age, sex, diagnosis (COPD vs. non-COPD), presence and grade of PH, and smoking status for the study population (n = 100) SD: standard deviation; COPD: chronic obstructive pulmonary disease; PH: pulmonary hypertension

Variables		Values
Age (years), mean ± SD		62 ± 12.1
Sex, n (%)	Male	68 (68%)
	Female	32 (32%)
Diagnosis, n (%)	COPD	75 (75%)
	Non-COPD	25 (25%)
PH, n (%)	Yes	48 (48%)
	No	52 (52%)
Grade of PH, n (%)	Mild	37 (75.5%)
	Moderate	8 (16.3%)
	Severe	4(8.2%)
Smoker, n (%)	Yes	57 (57%)
	No	43 (43%)

Significant differences were noted between the COPD and non-COPD groups. COPD patients were older (65.71 ± 8.3 vs. 51.8 ± 11.52 years; p < 0.001). Hematological parameters showed significantly higher RBC counts (4.67 × 10⁶ vs. 3.33 × 10⁶; p < 0.001), PLT (318,720 ± 99,905 vs. 264,500 ± 112,661; p = 0.018), and RDW (15.4 ± 0.92 vs. 11.74 ± 1.37; p < 0.001) in COPD patients. PLR was also significantly elevated (253.8 ± 86.6 vs. 126.5 ± 79.4; p = 0.010). Lymphocyte counts were significantly lower in COPD patients (1,352.6 ± 483.9 vs. 2,253.4 ± 867.6; p < 0.001). HB levels were slightly higher in COPD patients (14.65 ± 2.01 vs. 13.42 ± 1.53; p = 0.030). Pulmonary function testing revealed significantly lower FEV1 (47.93 ± 15.76 vs. 59.2 ± 17.16; p = 0.009), FEV1/FVC ratio (0.58 ± 0.09 vs. 0.8 ± 0.05; p < 0.001), FEF_25-75_ (29.6 ± 18.5 vs. 51.8 ± 27.2; p < 0.001), and DLCO (69 ± 17.1 vs. 93 ± 10; p < 0.001) in the COPD group. FVC values were not significantly different (p = 0.630). Chi-square analysis revealed statistically significant associations between COPD and sex (χ² = 19.9; p < 0.001), smoking status (χ² = 27.5; p < 0.001), and presence of PH (χ² = 27.0; p < 0.001). PH was exclusively observed in the COPD group. Grading of PH was also reported only in COPD patients, with 37 patients showing mild PH and 12 with moderate/severe PH, as shown in Table 2.

**Table 2:** Comparison of clinical, demographic, and blood parameters between COPD and non-COPD groups This table compares various clinical and demographic parameters between patients with COPD and those without COPD. Data are presented as mean ± SD or as n for categorical variables, with corresponding statistical and p values for comparisons SD: standard deviation; COPD: chronic obstructive pulmonary disease; FVC: forced vital capacity; FEV1: forced-expiratory volume in one second; FEF 25-75: forced-expiratory flow between 25% and 75% of vital capacity; DLCO: diffusing capacity of the lung for carbon monoxide; NA: not applicable; PDW: platelet distribution width; PCT: plateletcrit; MPV: mean platelet volume; PLT: platelet count; RBC: red blood cell; PLR: platelet-to-lymphocyte ratio; RDW: red cell distribution width; HB: hemoglobin; PH: pulmonary hypertension

Parameter	COPD, mean ± SD or n	non-COPD, mean ± SD or n	t value or chi-square (χ²) value	p value
Age (years)	65.71 ± 8.3	51.8 ± 11.52	19.9	<0.001
Sex (female/male)	15/60	17/8	NA	<0.001
PH (no/yes)	27/48	25/0	27.0	<0.001
Smoker (no/yes)	21/54	22/3	27.5	<0.001
Grading of PH (mild PH vs. mod/severe PH)	37/12	0/0	NA	NA
PDW (fL)	10.23 ± 1.64	11.16 ± 1.64	-2.47	0.015
PCT (%)	0.3 ± 0.08	0.26 ± 0.07	2.31	0.023
MPV (fL)	10.03 ± 1.73	10.14 ± 1.27	-0.28	0.780
PLT (cells/mm^3^)	318,720 ± 99,905.2	264,500 ± 112,661.7	2.40	0.018
RBC counts (cells/mm^3^)	4,670,000 ± 907,446.7	3,330,000 ± 1,260,000	5.80	<0.001
Lymphocyte counts (cells/mm^3^)	1,352.6 ± 483.9	2,253.4 ± 867.6	-6.49	<0.001
PLR	253.8 ± 86.6	126.5 ± 79.4	6.48	0.010
RDW (%)	15.4 ± 0.92	11.74 ± 1.37	15.08	<0.001
HB (g/dL)	14.65 ± 2.01	13.42 ± 1.53	2.78	0.030
FVC (%)	58.92 ± 14.84	57.96 ± 18.02	0.27	0.630
FEV1 (%)	47.93 ± 15.76	59.2 ± 17.16	-3.03	0.003
FEV1/FVC	0.584 ± 0.09	0.8 ± 0.050	-11.48	<0.001
FEF 25-75 (%)	29.56 ± 18.5	51.80 ± 27.2	-5.06	<0.001
DLCO (%)	69 ± 17.1	93 ± 10	-5.96	<0.001

Patients with COPD and confirmed PH (n = 49) were further categorized into mild (n = 37), moderate (n = 8), and severe (n = 4) PH groups. A comparative analysis across these subgroups revealed several clinically meaningful trends. Age increased progressively with PH severity, ranging from 63.3 ± 7.4 years in the mild group to 76.4 ± 8.6 years in the severe group (p = 0.046), suggesting a potential age-related worsening of vascular pathology. Among hematological markers, RDW demonstrated a statistically significant increase across the groups (15.5 ± 0.5 in mild, 16.6 ± 0.7 in moderate, and 16.85 ± 0.17 in severe; p < 0.001). This indicates increasing heterogeneity in red cell size distribution, possibly reflecting worsening hypoxemia or systemic inflammation in advanced PH. Similarly, PLR rose with disease severity (273.8 ± 82.4 in mild, 291.9 ± 62.3 in moderate, and 391.2 ± 79.2 in severe; p = 0.040), indicating progressive immune and inflammatory imbalance with advancing PH. While platelet indices, such as PDW, MPV, PCT, and PLT, exhibited a numerical increase from mild to severe PH, none of these reached statistical significance (p > 0.05); however, the trend may warrant further exploration in a larger cohort. DLCO, a marker of alveolar-capillary gas exchange, showed a significant inverse correlation with PH severity, declining from 67.6 ± 8.2 in mild PH to 54.6 ± 15.1 in moderate and 45.3 ± 12.6 in severe PH (p = 0.025). This finding reinforces the concept that worsening PH is associated with impaired pulmonary vascular perfusion and diffusion capacity. In contrast, spirometric values including FEV1, FVC, FEV1/FVC, and FEF_25-75 _did not differ significantly among PH severity groups (all p > 0.5), suggesting that while airflow obstruction is a hallmark of COPD, it may not directly correlate with PH progression. HB levels and lymphocyte counts also showed nonsignificant variation across groups. These results imply that RDW, PLR, age, and DLCO may serve as more reliable indicators of PH severity in COPD patients than conventional spirometric values or basic platelet indices, as shown in Table 3.

**Table 3:** Comparison of clinical and blood parameters across mild, moderate, and severe PH groups This table presents a comparison of clinical and blood parameters among patients stratified by the severity of PH (mild PH, moderate PH, and severe PH). Data are expressed as mean ± SD, with p values indicating statistical significance between the groups PH: pulmonary hypertension; SD: standard deviation; PDW: platelet distribution width; PCT: plateletcrit; MPV: mean platelet volume; PLT: platelet count; RBC: red blood cell; PLR: platelet-to-lymphocyte ratio; RDW: red cell distribution width; HB: hemoglobin; FVC: forced vital capacity; FEV1: forced-expiratory volume in one second; FEF 25-75: forced-expiratory flow between 25% and 75% of vital capacity; DLCO: diffusing capacity of the lung for carbon monoxide

Parameter	Mild PH, mean ± SD	Moderate PH, mean ± SD	Severe PH, mean ± SD	f value	p value
Age (years)	63.3 ± 7.36	66 ± 9.47	76.4 ± 8.6	4.84	0.046
PDW (fL)	10.15 ± 1.85	10.34 ± 1.56	10.35 ± 1.80	0.05	0.951
PCT (%)	0.3 ± 0.1	0.3 ± 0.11	0.38 ± 0.06	3.83	0.070
MPV (fL)	10.22 ± 2.12	10.64 ± 1.91	10.70 ± 1.71	0.23	0.804
PLT (cells/mm^3^)	330,054.1 ± 102,302.4	342,125 ± 102,733.2	448,400.0 ± 139,211.3	1.56	0.266
RBC counts (cells/mm^3^)	4.71 × 10⁶ ± 0.78 × 10⁶	4.33 × 10⁶ ± 1.90 × 10⁶	5.18 × 10⁶ ± 0.36 × 10⁶	2.41	0.144
Lymphocyte count (cells/mm^3^)	1,273.4 ± 437.3	1,272.5 ± 612.1	1,180 ± 462.3	0.09	0.919
PLR	273.8 ± 82.4	291.9 ± 62.3	391.2 ± 79.2	4.67	0.040
RDW (%)	15.55 ± 0.54	16.55 ± 0.69	16.85 ± 0.17	52.88	<0.001
HB (g/dL)	15.05 ± 1.72	14.8 ± 2.43	17.18 ± 1.85	2.30	0.175
FVC (%)	55.6 ± 10.98	58.5± 8.62	52.75 ± 11.7	0.47	0.644
FEV1 (%)	43.87 ± 12.1	43.88 ± 14	41.75 ± 8.22	0.10	0.908
FEV1/FVC	0.579 ± 0.0773	0.549 ± 0.105	0.533 ± 0.077	0.72	0.517
FEF 25-75 (%)	27.68 ± 14.2	28.3 ± 16.7	33.00 ± 24.18	0.09	0.919
DLCO (%)	67.65 ± 8.2	54.57 ± 15.13	45.3 ± 12.6	7.24	0.025

## Discussion

Our study investigated the blood indices values between COPD and non-COPD participants and their potential for predicting the development of PH in COPD patients. Our study findings revealed distinct hematological profiles associated with COPD and identified specific indices that correlate with PH severity, offering insights into disease pathophysiology and potential diagnostic utility.

 A meta-analysis study done by Zhang et al. [21] reviewed 38 standard articles around the globe for the prevalence of PH in COPD patients and found the pooled prevalence of PH to be about 39.2%, and the pooled prevalence of each grade, mild, moderate, and severe PH, was 30.2%, 10%, and 7.2%, respectively. Our study had a prevalence of PH in 64% of the COPD patients; mild, moderate, and severe PH were observed in 49%, 16%, and 5% respectively. This variability can be explained by the fact that different studies have different criteria for diagnosing PH. According to ESC/ERS guidelines [21], the gold standard for diagnosing PH is the right heart catheterization (RHC) value of mean pulmonary arterial pressure >20 mmHg at rest, but a better, noninvasive, and safer method of diagnosing PH would be to do a transthoracic echocardiography to look for features of PH. Hence, it was used in our study.

Red cells and platelets are involved in the pathophysiology of not only PH in COPD but also in the development of COPD itself [1,22]. Moreover, according to the study by Zinellu et al. [4] and Muñoz-Esquerre et al. [10], platelet pathway activation is strongly associated with the exacerbation of COPD, worsening the prognosis of the disease process and increasing the mortality rate. Platelet pathway activation is, in turn, reflected in the platelet indices, affecting the morphology and function of the platelets. Red cells are associated with oxygen transport and inflammation, as described by Tariq et al. [23], whose study showed that lower levels of HB, hematocrit, and mean corpuscular HB concentration, alongside elevated RDW, are linked to poorer prognosis, increased mortality, and higher hospitalization rates in COPD patients. RBC indices are therefore considered crucial prognostic indicators for assessing disease severity, guiding treatment, and monitoring follow-up in COPD management. Our study demonstrated that COPD patients had significantly lower lymphocyte counts (p < 0.01), higher PLR (p < 0.01), increased RDW (p < 0.01), decreased PDW (p < 0.01), and elevated PLT counts (p < 0.01) compared to non-COPD patients. However, MPV and PCT did not show any statistically significant correlation.

Some previous studies [14,24] have shown that COPD patients who developed PH had higher RDW values (>15.5%). According to Venkatesh et al. [25], along with RDW, MPV and PDW also correlated positively with PH in COPD patients, and these indices increased with the severity of PH in COPD. Xie et al. [26] demonstrated that RDW has not only diagnostic value but also prognostic value for PH in COPD patients. Our study showed that only RDW had predictive value for PH in COPD patients, and it increased with the grade of PH, while other parameters, including MPV and PDW, showed no correlation.

PLR has been studied previously [4,27,28] as a marker for exacerbation of COPD, a predictor of inhospital mortality in intensive care settings, and a predictor of heart failure. Our study has shown that PLR is higher in COPD patients when compared with non-COPD participants. It has also been shown to be significantly correlated with the development of PH in COPD, and it increases with an increase in the severity of PH.

Our study also correlated spirometry values with the severity of PH and found a positive correlation with DLCO values, but no significant changes in FEV1 and FEF_25-75_ were noted. This can be explained by the alveolar capillary membrane disruption, causing changes in pulmonary vasculature [29].

To our knowledge, our study is the first to comprehensively correlate spirometry values with all red cell and platelet indices in patients with PH and COPD. This integrated approach aims to better understand their collective association with PH in COPD. Our findings contribute novel insights to the existing literature.

Limitations

Despite its strengths, this study has several limitations. The sample size, although diverse, may not be representative of all COPD populations, which could potentially limit the generalizability of the findings. Potential confounding factors such as underlying comorbidities (e.g., cardiovascular disease) and the inflammatory burden from smoking may still influence the blood indices studied. Future longitudinal studies with larger, multicentric cohorts and multivariate analysis are needed to confirm these findings and better account for such confounders. Transthoracic 2D echocardiography, though a noninvasive method of diagnosing PH, tends to overestimate the presence of PH in some cases; hence, RHC is regarded as the gold standard for diagnosing PH.

## Conclusions

In conclusion, blood markers, namely PLR and RDW, are significantly associated with the presence of PH and predict the severity of PH in COPD patients. PLR and RDW are two promising blood markers for predicting PH in COPD patients, which can be easily obtained from a simple complete blood hemogram, making them a cost-effective and readily available option in low-resource settings for early suspicion, early referral, and timely intervention. Further research, particularly prospective studies, is needed to confirm their predictive value and explore their integration into routine clinical practice for early diagnosis and management of PH in COPD.
